# Nonalcoholic Fatty Liver Disease and Type 2 Diabetes Mellitus: A Bidirectional Relationship

**DOI:** 10.1155/2020/6638306

**Published:** 2020-12-28

**Authors:** Cristina M. Muzica, Catalin Sfarti, Anca Trifan, Sebastian Zenovia, Tudor Cuciureanu, Robert Nastasa, Laura Huiban, Camelia Cojocariu, Ana-Maria Singeap, Irina Girleanu, Stefan Chiriac, Carol Stanciu

**Affiliations:** ^1^Department of Gastroenterology, Grigore T. Popa University of Medicine and Pharmacy, Iasi 700115, Romania; ^2^St. Spiridon Emergency Hospital, Iasi 700115, Romania

## Abstract

Worldwide, the leading cause of chronic liver disease is represented by nonalcoholic fatty liver disease (NAFLD) which has now become a global epidemic of the 21st century, affecting 1 in 4 adults, and which appears to be associated with the steadily increasing rates of metabolic syndrome and its components (obesity, type 2 diabetes mellitus (T2DM), and dyslipidemia). NAFLD has been reported to be associated with extrahepatic manifestations such as cardiovascular disease, T2DM, chronic kidney disease, extrahepatic malignancies (e.g., colorectal cancer), endocrine diseases (e.g., hypothyroidism, polycystic ovarian syndrome, psoriasis, and osteoporosis), obstructive sleep apnea, and iron overload. The prevalence of NAFLD is very high, affecting 25–30% of the world population and encloses two steps: (1) nonalcoholic fatty liver (NAFL), which includes steatosis only, and (2) nonalcoholic steatohepatitis (NASH) defined by the presence of steatosis and inflammation with hepatocyte ballooning, with or without fibrosis which can progress to liver fibrosis, hepatocellular carcinoma, and liver transplantation. Current data define a more complex relationship between NAFLD and T2DM than was previously believed, underlining a bidirectional and mutual association between the two entities. This review aims to summarize the current literature regarding the incidence of T2DM among patients with NAFLD and also the prevalence of NAFLD in T2DM patients, highlighting the recent key studies. Clinicians should screen, diagnose, and treat T2DM in patients with NAFLD in order to avoid short- and long-term complications.

## 1. Introduction

The incidence rate of chronic liver diseases increased through the years with a worrying rise of liver-related morbidity and mortality rates worldwide [1]. One of the major causes of chronic liver diseases is represented by nonalcoholic fatty liver disease (NAFLD) which has now become a global epidemic affecting 1 in 4 adults, with an estimated prevalence between 25% and 30%, and appears to be associated with the steadily increasing rates of metabolic syndrome (MetS) and its components (obesity, type 2 diabetes mellitus (T2DM), and dyslipidemia) [1–3]. The mandatory feature of NAFLD is the presence of liver steatosis (LS) in the absence of other causes of chronic liver disease [4]. Although initially NAFLD was considered as the hepatic manifestation of MetS, there is now clear evidence that NAFLD is a key driver in MetS and hepatic involvement is only one component of systemic multiorgan involvement [5, 6]. NAFLD encloses two distinct conditions with different histologic features and prognosis: (1) nonalcoholic fatty liver (NAFL), which includes steatosis only, and (2) nonalcoholic steatohepatitis (NASH) characterized by steatosis and inflammation with hepatocyte ballooning, with or without fibrosis, which can progress to liver fibrosis (LF), cirrhosis, hepatocellular carcinoma (HCC), and liver transplantation [7]. The prevalence of NASH among NAFLD patients ranges from 10% to 59% in patients who underwent liver biopsy [1], meaning that millions and millions of people worldwide are at risk of cirrhosis and its complications. Even more alarming is that the continuously increasing rates of MetS and its components parallel the rising prevalence of NASH, with obesity, T2DM, and MetS being the most important risk factors [8, 9].

Growing evidence clearly shows that NAFLD is a multiorgan disease, supporting a strong link between NAFLD and cardiovascular diseases (CVDs), T2DM, chronic kidney disease (CKD), extrahepatic malignancies (eg., colorectal cancer), obstructive sleep apnea (OSA), and various endocrinopathies (e.g., thyroid dysfunction, polycystic ovarian syndrome (PCOS), osteoporosis, psoriasis, hypothyroidism, and iron overload) [10, 11]. Although the primary site of NAFLD is the liver, the most common causes of mortality are CVDs, followed by extrahepatic malignancies such as colorectal cancer and then liver-related complications (cirrhosis and HCC) [5, 6, 12]. Considering the high clinical and economic burden of NAFLD, the main point in the management of these patients is an early acknowledgement of both hepatic and extrahepatic manifestations and their subsequent complications [11, 13, 14]. Current data outline a more complex relationship between NAFLD and T2DM than was previously thought, pointing out a bidirectional and mutual association between the two entities. Thus, clinicians should screen, diagnose, and treat T2DM in patients with NAFLD in order to avoid short- and long-term complications. Herein, this review aims to summarize the current literature regarding the incidence of T2DM among patients with NAFLD and also the prevalence of NAFLD in T2DM patients, highlighting recent key studies.

## 2. Relation between NAFLD and T2DM

Current data reveal a more complex relationship between NAFLD and T2DM than was previously believed, highlighting a bidirectional and mutual association between the two entities [11, 15, 16]. Considering that NAFLD and T2DM have similar physiopathological pathways, one can precede and/or promote the other [10, 17].

### 2.1. T2DM in Patients with NAFLD

NAFLD is associated with lipotoxicity which is secondary to the accumulation of triglyceride-derived toxic metabolites in the liver, pancreas, and muscles, which leads to the activation of the inflammation cascade and insulin resistance [18, 19]. The hepatic insulin resistance associated with NAFLD is the key driver for the development of T2DM among these patients.

The prevalence of T2DM in patients with NAFLD depends on the severity of NAFLD starting from 9.8% in mild NAFLD to 17.8% in moderate to severe NAFLD [20–22]. Although NAFLD is considered an independent risk factor for developing T2DM, with a 2-fold incidence increase in these individuals, patients with NASH have an up to threefold higher risk of developing incident T2DM compared with those with simple steatosis [20, 23, 24].

Despite evidence from several studies which demonstrated that high levels of NAFLD's surrogate markers—gamma-glutamyl transferase and alanine aminotransferase (ALT)—were associated with a high incidence of T2DM, the predictive value of these biologic parameters is limited due to the possibility of normal levels among these patients [25–29]. As for ultrasonography-defined NAFLD (liver steatosis ≥20%) [30, 31], recent data showed a twofold to fivefold increased risk of T2DM [32].

Many large-population-based retrospective studies with a follow-up period of 3 to 6.2 years showed an increased incidence risk of T2DM among patients diagnosed with NAFLD (Table 1) [33–37, 42, 43]. Shibata et al. achieved a 4-year follow-up in 3189 patients among whom 1138 had NAFLD at baseline and reported an incidence of 1.8% vs. 8.1% in the non-NAFLD patients compared with those with NAFLD [33]. Similarly, 4 other retrospective studies which included Asian cohorts without T2DM, demonstrated that NAFLD was significantly associated with a high incidence of T2DM during follow-up [34–37]. In a similar manner, prospective studies which evaluated the prevalence of T2DM among patients with NAFLD have also reported an increased risk associated with baseline NAFLD [38–41]. Results from an Israeli cohort of 141 nondiabetic participants among which 24.8% had NAFLD, followed up for an average period of 6.08 ± 0.7 years, demonstrated a higher incidence of prediabetes and/or T2DM in patients with NAFLD than in subjects without NAFLD (74.3% vs. 48.1%) [39]. In a recent retrospective study conducted by Liu et al. which included 18,507 nondiabetic subjects, the prevalence of NAFLD was 18.77% with a 5-year T2DM incidence of 2.44%. The authors concluded that patients with baseline NAFLD had a higher risk incidence of T2DM, with an adjusted relative risk of 1.672 [43].

### 2.2. NAFLD in Patients with T2DM

Once established, T2DM may promote the progression to NASH and become an independent risk factor for chronic liver disease, including cirrhosis and HCC [10]. It has been reported that the presence of NAFLD among patients with T2DM determined a 2.2-fold risk increase in all-cause mortality, compared with those without NAFLD [15]. The estimated prevalence of NAFLD among patients with T2DM is about 75%, which is more than the prevalence in the general population [1].

Evidences from several recent cohort studies highlighted an increased incidence of NAFLD among patients diagnosed with T2DM, with an estimated prevalence ranging between 41.6% and 86% (Table 2) [8, 44–49]. For instance, Sporea et al. conducted a prospective study in which 534 patients diagnosed with T2DM were included. Patients with other causes of steatosis were excluded, and LF and steatosis were quantitatively assessed using vibration controlled transient elastography and the controlled attenuation parameter. The authors found an estimated prevalence of NAFLD of 76.1%. Moreover, severe steatosis was detected in 60.3%, while advanced LF had a prevalence of almost 20% [50]. Two other retrospective studies reported a similar prevalence of NAFLD among T2DM patients [45, 46].

## 3. Management of Patients with NAFLD and T2DM

The reciprocal relationship between T2DM and NAFLD leads to the progression of LF and is secondary to the development of liver-related complications with high morbidity and mortality rates. In order to avoid systemic multicollateral damage, it seems appropriate to screen patients with NAFLD for T2DM, and vice-versa. According to current European Guidelines, screening of T2DM in patients with NAFLD is mandatory, and it consists of random blood glucose or hemoglobin A1C [51]. Furthermore, screening for NAFLD and LF is recommended by the American Diabetes Association in patients diagnosed with T2DM which have elevated liver enzymes (ALT) or LS, while the American Association Society of Liver Disease is not in favour of routine screening in these patients [52, 53].

## 4. Conclusion

In light of current evidence, the clinical burden of NAFLD is not limited to liver-related complications, but is in fact, related to its extrahepatic manifestations such as CVD, T2DM, CKD, extrahepatic malignancies, OSA, and various endocrinopathies, with increased mortality rates. Our review highlights that the components of NAFLD (NASH and NAFL) lead to insulin resistance and T2DM through various physiopathological pathways, but also T2DM may promote the progression to NASH as an independent risk factor. Thus, clinicians should be aware of these NAFLD-related extrahepatic manifestations considering that an early acknowledgement of T2DM is the key point in the management of these patients.

## Figures and Tables

**Table 1:** Studies which assessed T2DM incidence among patients with NAFLD.

Ref.	Study design and period of surveillance	NAFLD assessment	Cases with T2DM; % in NAFLD vs. non-NAFLD
Shibata et al. 2007 [33]	Retrospective cohort study; *n* = 3,189 (33.6% with NAFLD); male Japanese; 4 years	Liver ultrasonography	*n* = 109 incident cases; 1.8% vs. 8.1%
Kim et al. 2008 [34]	Retrospective cohort study; *n* = 5,372 (33.3% with NAFLD); South Korean subjects without diabetes; 5 years	Liver ultrasonography	*n* = 234 incident cases; 2.3% vs. 8.5%
Bae et al. 2011 [35]	Retrospective study; *n* = 7,849 (29.2% with NAFLD); subjects without diabetes; 5 years	Liver ultrasonography	*n* = 435 incident cases; 3.7% vs. 9.9%
Sung et al. 2012 [36]	Retrospective cohort study; *n* = 12,853 (27.6% with NAFLD); subjects without diabetes; 5 years	Liver ultrasonography	*n* = 223 incident cases; 0.8% vs. 4.3%
Kasturirante et al. 2013 [37]	Retrospective cohort study; *n* = 2,276 (40.7% with NAFLD); individuals without diabetes; 3 years	Liver ultrasonography	*n* = 242 incident cases; 10.5% vs. 19.7%
Park et al. 2013 [38]	Prospective cohort study; *n* = 25,232 (35% with NAFLD); men without diabetes; 5 years	Liver ultrasonography	*n* = 2,108 incident cases; 7% in no steatosis group vs. 17.8% in moderate to severe steatosis group
Zelber-sagi et al. 2013 [39]	Prospective cohort study, *n* = 213; without known liver disease and alcohol abuse; 7 years	Liver ultrasonography	*n* = 106 incident cases with NAFLD
Chen et al. 2016 [40]	Prospective cohort study; *n* = 6,542; Chinese subjects without diabetes; 6 years	Liver ultrasonography	*n* = 368 incident cases
Li et al. 2017 [41]	Prospective cohort study; *n* = 18,111 (31.8% with NAFLD); Chinese subjects without diabetes without known chronic liver diseases; 4.6 years follow-up	Liver ultrasonography	*n* = 1,262 incident cases; 4.6% in non-NAFLD group vs. 18.1 in moderate to severe NAFLD group
Ma et al. 2017 [42]	Retrospective cohort study; *n* = 1,051 (17.8% with NAFLD); US individuals without diabetes without known chronic liver diseases; 6.2 years	Liver tomography and ultrasonography	*n* = 64 incident cases
Liu et al. 2017 [43]	Retrospective study, *n* = 18,507; men without diabetes; 5 years	Liver ultrasonography	*n* = 453 incident cases

NAFLD, nonalcoholic fatty liver disease; T2DM, type 2 diabetes mellitus.

**Table 2:** Studies which assessed NAFLD prevalence among patients with T2DM.

Ref.	Study design; follow-up; and population	Diagnostic method of NAFLD	Prevalence of NAFLD
Williamson et al. 2011 [8]	Retrospective study; *n* = 918; 1 years	Ultrasound	42,6%
Lv et al. 2013 [44]	Prospective cohort study; *n* = 1217; 4 years	Ultrasound	61%
Silaghi et al. 2015 [45]	Retrospective cohort study; *n* = 336; N/A	Ultrasound	86%
Mantovani et al. 2016 [46]	Retrospective cohort study; *n* = 330; 2 years	Ultrasound	72,1%
Guo et al. 2017 [47]	Prospective cohort study; *n* = 8571; 9 years	Ultrasound	50,6%
Yi et al. 2017 [48]	Prospective cohort study; *n* = 3861; 1 year	Ultrasound	45,4%
Ding et al. 2017 [49]	Prospective cohort study; *n* = 1648; 1 year	Ultrasound	41,6%
Sporea et al. 2020 [50]	Prospective cohort study; *n* = 534; N/A	Ultrasound	76,1%

NAFLD, nonalcoholic fatty liver disease; T2DM, type 2 diabetes mellitus; N/A, not available.
